# Assessment of ultraviolet B–blocking effects of weekly disposable contact lenses on corneal surface in a mouse model

**Published:** 2013-05-29

**Authors:** David Pei-Cheng Lin, Han-Hsin Chang, Li-Chien Yang, Tzu-Ping Huang, Hsiang-Jui Liu, Lin-Song Chang, Chien-Hsun Lin, Bo-Yie Chen

**Affiliations:** 1 School of Medical Laboratory and Biotechnology, Chung Shan Medical University, Taichung, Taiwan, ROC; 2 Department of Ophthalmology, Chung Shan Medical University Hospital, Taichung, Taiwan, ROC; 3 School of Nutrition, Chung Shan Medical University, Taichung, Taiwan, ROC; 4 Department of Optometry, Hsin Sheng College of Medical Care and Management, Taoyuan, Taiwan, ROC; 5 School of Optometry, Chung Shan Medical University, Taichung, Taiwan, ROC; 6 Department of Optometry, Mackay Medicine, Nursing and Management College, Taipei, Taiwan, ROC; 7 Division of Vision Sciences, Institute of Biomedical Sciences, Chung Shan Medical University, Taichung, Taiwan, ROC; 8 Core Laboratory of Vision Protection and Biotechnology, Chung Shan Medical University, Taiwan, ROC; 9 Department of Optometry, Yuanpei University, Hsinchu, Taiwan, ROC

## Abstract

**Purpose:**

Weekly disposable soft contact lenses have been widely used recently, but their shield effects against ultraviolet (UV) irradiation remain to be evaluated. This study investigated the bioprotective effects of several weekly soft contact lenses against UVB irradiation on the corneal surface in a mouse model.

**Methods:**

Fifty ICR mice were randomly divided into five groups: (1) blank control, (2) exposed to UVB without contact lens protection, (3) exposed to UVB and protected with Vifilcon A contact lenses, (4) exposed to UVB and protected with Etafilcon A contact lenses, and (5) exposed to UVB and protected with HEMA+MA contact lenses. The exposure to UVB irradiation was performed at 0.72 J/cm^2^/day after anesthesia for a 7-day period, followed by cornea surface assessment for smoothness, opacity, and grading of lissamine green staining. Tissue sections were prepared for hematoxylin and eosin staining and immunohistochemical detection by using antibodies against myeloperoxidase, cytokeratin-5, P63, Ki-67, nuclear factor-kappa B (p65), cyclooxygenase-2, Fas L, and Fas.

**Results:**

The results showed impaired corneal surface with myeloperoxidase^+^ polymorphonuclear leukocyte infiltration into the stroma after UVB exposure, in contrast to the intact status of the blank controls. The corneas with Etafilcon A and HEMA+MA contact lenses maintained more cells positive for cytokeratin-5, P63, and Ki-67 compared to those with Vifilcon A or without contact lens protection. Furthermore, less proinflammatory factors, including nuclear factor-kappa (p65), cyclooxygenase-2, Fas L, and Fas, were induced in the corneas protected by Etafilcon A and HEMA+MA.

**Conclusions:**

This study demonstrated various protective effects of weekly disposable contact lenses against UVB irradiation. The mouse model used in the present study may be used extensively for in vivo assessment of UV shield efficacy.

## Introduction

The cornea contributes a transparent front surface to the eye and plays a pivotal role in providing correct refractive power for clear vision. To fulfill this function, a cornea has to allow light to pass through and therefore can undergo phototoxic damage caused by ultraviolet irradiation. Previous literature has shown that excessive ultraviolet (UV) exposure leads to various symptoms on the ocular surface [1], including exfoliation of the corneal epithelium, reduced transparency and smoothness [2], decreased visual acuity, inflammation, edema, eye redness, and burning-like pain [3,4]. These symptoms, often collectively called photokeratitis or ultraviolet keratitis, indicate significant damage to the eye [3,5]; this damage may not be limited to the corneal epithelium. UV irradiation can go deeper through the epithelial layer and induce inflammatory responses that span the full thickness of the cornea [3,6-8]. The cellular and molecular mechanisms underlying ultraviolet photokeratitis have been extensively investigated in recent years [9-12]. Notably, progression of the disease involves various factors such as interleukins, cytokines, matrix metalloproteinases (MMPs), nuclear factor-kappa B (NF-κB), cyclooxygenase-2 (COX-2), inducible nitric oxide synthase (iNOS), iNOS-derived nitric oxide (NO⋅), reactive oxygen species (ROS), and malondialdehyde (MDA).

Since ultraviolet photokeratitis has to be avoided, correction of refraction errors with simultaneous prevention of UV-induced damage has been a pivotal concern in the lens design industry. UV-blocking lenses can filter out straight UV transmission to the central cornea but cannot completely protect against peripheral UV light that may also focus on the central cornea [13]. In contrast, UV-blocking contact lenses may provide better protection against peripheral light [14,15]. Thus, clinical indications for UV-blocking contact lenses include hypersensitivity to UV light, aphacia, or professionals whose work involves routine eye exposure to high levels of UV light. Nevertheless, although contact lenses have been available for decades [16], only some disposable contact lenses are made of different fabrication materials with various UV absorption characteristics [17-19]. Until recently, only a few reports addressed the UV-blocking effects of contact lenses under in vivo conditions. For example, the UV protection efficacy of Senofilcon A hydrogel contact lenses in rabbit eyes was reported, showing protection against UVB-induced damage to the cornea and lens and reduction in UVB-mediated activation of matrix metalloproteinases [17,18]. Most recently, the Senofilcon A silicon hydrogel contact lens was assessed for protective effects against UVB-induced membrane lipid peroxidation and cellular DNA damage in the mouse cornea and retina [19]. The protective effects of many other soft hydrogel contact lenses remain to be evaluated under in vivo conditions, and the underlying biologic effects need to be further elucidated.

We previously reported that, after excessive exposure to UVB, the mouse cornea showed significant damage such as corneal ulcer, epithelial exfoliation, and polymorphonuclear leukocyte (PMN) infiltration [9]. The mouse model is applicable for in vivo assessment of UVB-blocking efficacy by different contact lenses. In this study, we used the mouse model to assess UVB-protective effects of three weekly disposable contact lenses made of different fabrication materials.

## Methods

### Animals

Fifty 6-week-old female ICR mice were purchased from the National Laboratory Animal Center (Taipei, Taiwan). The mice weighed about 25 g on arrival. They were fed ad libitum and kept under standard conditions with a 12 h:12 h light-dark cycle. All mice were examined with a dissecting microscope (SMZ 1500; Nikon, Tokyo, Japan) before the experiments. Only mice without anomalies in the anterior segment (cornea, anterior chamber, iris, or lens) were included in the experiments.

### Contact lenses and ultraviolet B exposure

**Figure 1:**
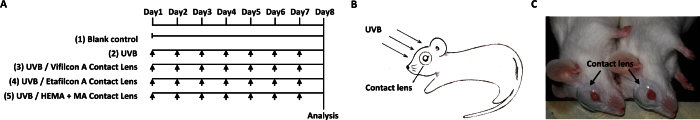
Experimental groups and setting of contact lens protection. **A**: Daily ultraviolet B (UVB) light exposure (indicated by arrows) was performed from day 1 to day 7, with or without weekly disposable soft hydrogel contact lenses, respectively. No contact lens was given to the UVB group or the blank control group. **B**: The diagrammatic illustration shows a mouse being anesthetized with an eye exposed to UVB and covered by a contact lens. **C**: An actual experimental preparation of mouse eyes covered with contact lenses is demonstrated.

The mice were randomly split into five groups (Figure 1A), including (1) blank control without UVB exposure, (2) with UVB exposure but without contact lenses, (3) with UVB exposure and Vifilcon A contact lenses (Focus 1–2 Week, CIBA Vision Corp., Duluth, GA), (4) with UVB exposure and Etafilcon A contact lenses (ACUVUE2, Johnson & Johnson Vision Care Inc., Jacksonville, FL), and (5) with UVB exposure and HEMA+MA contact lenses (TICON 55 UV, St. Shine Optical Co. Ltd., New Taipei City, Taiwan). Following anesthesia with intraperitoneal sodium pentobarbital injection (45 mg/kg bodyweight), the mouse eyes of groups (3) to (5) were covered with the respective contact lenses and exposed to UVB light (CN-6, Vilber Lourmat, Moune La Vallee, Cedex, France). The UVB source was 8 mW/cm^2^, and the exposure time was set at 90 s to reach a total amount of 0.72 J/cm^2^/day, for a 7-day period in a darkroom (Figure 1B,C) [9,20]. The wavelength of the UVB light ranged between 280 nm and 320 nm with a peak at 312 nm, as measured with a UV detector (VLX-3W, Vilber Lourmat) from the same company. The UVB transmittance (mean±standard deviation [SD]) at day 1 (before UVB exposure) and day 7 (after UVB exposure) for each type of contact lens used in this study were 42.17±2.75% (before) and 48.19±4.38% (after) for Vifilcon A, 0.86±0.12% (before) and 1.43±0.24% (after) for Etafilcon A, and 1.39±0.14% (before) and 1.64±0.15% for HEMA+MA. During all UVB exposure procedures, there was no mouse eye closure due to the effect of the anesthesia. After each daily UVB exposure, the mice in groups (2) to (5) were allowed to recover for 10 min, during which 0.9% saline eye drops were applied several times to avoid dryness on the eye surface. The mice were then transferred to their original cages set under normal room light. The mice in the blank control group were treated in a similar manner except UVB exposure. Each weekly disposable soft contact lens used in this study was repetitively exposed to UVB seven times. When not in use, the contact lenses were carefully sealed in the preservation buffer and kept under room temperature. Care was taken to avoid bacterial contamination, and thorough rinses in 0.9% saline were performed to avoid carryover of the preservation buffer to the eye surface. All experiments were reviewed and approved by the Animal Care and Use Committee of Chung Shan Medical University and were performed in agreement with the Association for Research in Vision and Ophthalmology (ARVO) Resolution on the Use of Animals in Research.

### Scoring of corneal smoothness, opacity, and lissamine green staining

All mice were anaesthetized before assessment on day 8 (Figure 1A). One eye of each mouse was randomly selected to assess corneal smoothness. The other eye was then assessed for corneal opacity. The experimental procedures and the criteria for corneal smoothness scoring were applied following the protocols published previously [9]. Briefly, images of the cornea surface were taken with a stereoscopic zoom microscope equipped with a ring illuminator (SMZ 1500; Nikon). Based on the digital images, the corneal smoothness scores were determined by using a 5-point scale based on the number of distorted quadrants in the reflected ring: 0, no distortion; 1, distortion in one quadrant of the ring (3 clock h); 2, distortion in two quadrants (6 clock h); 3, distortion in three quadrants (9 clock h); 4, distortion in all four quadrants (12 clock h); and 5, severe distortion, in which no ring was recognized. For corneal opacity scoring, the images were scored from 0 (normal) to 4 (severe) in all corneas [9]. The criteria were 0, normal cornea; 0.5, mild haze seen only under the dissection microscope; 1, mild haze; 2, moderate haze with visible iris; 3, severe haze with invisible iris; and 4, severe haze with corneal ulceration. After corneal smoothness and opacity were scored, both corneas from each mouse were stained with 3 μl of 1% lissamine green (Sigma-Aldrich, St. Louis, MO). Images of lissamine green staining on the cornea surface were taken and scored according to a grading system based on the areas of stain in the cornea [9]. Briefly, the total area without punctuate staining was designated grade 0; grade 1, less than 25% of the cornea stained with scattered punctuate staining; grade 2, 25%–50% of the cornea stained with diffuse punctate staining; grade 3, 50%–75% of the cornea stained with punctuate staining and apparent epithelial defects; grade 4, more than 75% of the cornea stained with abundant punctuate staining and large epithelial defects. All scoring was performed by two observers without prior knowledge of the UVB exposure and study groups.

### Histopathological analysis and immunohistochemistry

Following assessment of corneal damage, the mice were euthanized with cervical dislocation. One of the mouse eyes, either the right eye or the left eye, was randomly selected and extracted. The extracted eyes, including the eyelids, were fixed in 4% formalin for at least 24 h, washed with 0.9% saline, and processed through ethanol and xylene solutions. The preparations were then embedded in paraffin, cut at 5-µm thickness, and mounted on glass slides following conventional procedures [9,21]. Hematoxylin and eosin (HE) staining was performed for histopathological examinations. For immunohistochemistry, the tissue sections were boiled in citrate buffer (pH 6.0) for 20 min for antigen retrieval and then incubated with one of the following antibodies: mouse anti-P63 (1/50, cat. no. sc-8431; Santa Cruz Biotechnology, Santa Cruz, CA), or rabbit anti-NF-κB-p65 (1/200, cat. no. E379; Epitomics, Burlington, CA), or rabbit anti-COX-2 (1/100, cat. no. ab15191; Abcam, Cambridge, MA), or rabbit antimyeloperoxidase (MPO) antibody (1/100, cat. no. RB-373; Labvision, Fremount, CA), or rabbit anticytokeratin 5 (CK-5) antibody (1/100, cat. no. NBP1–67613; Novus Biologicals, Littleton, CO), or rabbit anti-Ki-67 antibody (1/100, cat. no. NB110–89719; Novus Biologicals) or rabbit anti-Fas ligand (Fas L) antibody (1/50, cat. no. RB-9029; Labvision), or rabbit anti-Fas antibody (1/100, cat. no. NBP1–41407; Novus Biologicals). The preparations were then incubated with a horseradish peroxidase-conjugated secondary antibody (1/200), either antimouse or antirabbit immunoglobulin G (Jackson ImmunoResearch Laboratories, Inc., West Grove, PA). After incubation, the preparations were washed thoroughly, incubated in diaminobenzidine tetrahydrochloride solution for color detection, and counterstained with hematoxylin.

### Statistical analysis

All data were obtained from triple repeats. The data are presented as the means ± standard error of the means (SEMs) and were compared among groups. The corneal smoothness, opacity, and fluorescein staining scores were compared with the Kruskal–Wallis test. The corneal epithelium thickness and the number of infiltrative PNM, P63^+^ basal cells, and Ki-67^+^ cells were analyzed with the Mann–Whitney test. All statistical analyses were performed by using the SPSS program (SPSS, Inc., Chicago, IL).

## Results

### Reduction in corneal surface damage by ultraviolet-blocking contact lenses

**Figure 2:**
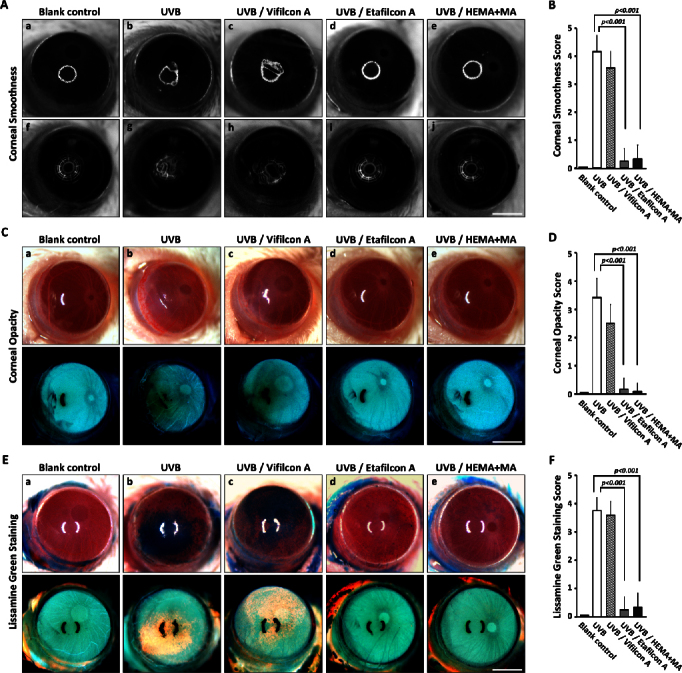
Representative photos for corneal surface evaluation among the experimental groups. **A**, **C**, and **E**: The corneal smoothness (**A**), corneal opacity (**C**), and lissamine green staining (**E**) among the five study groups were assessed for in vivo ultraviolet B (UVB) protective properties of contact lenses. The photos in the bottom row of C and E are the corresponding negative images. **B**, **D**, and **F**: Quantitative analysis of corneal smoothness (**B**), opacity (**D**), and lissamine green staining (**F**) among the five study groups (n=12 per groups) was performed. The results showed that all the scores were reduced in the groups with UVB-blocking contact lenses (Etafilcon A and HEMA+MA). The p<0.001 was determined with the Kruskal–Wallis test indicating significant difference from the UVB group. All scale bars represent 1.25 μm.

The effects of UVB irradiation on the corneal surface were first examined. The regularity of a single or multiple ring-shaped light reflected off the cornea surface was used to evaluate corneal epithelial smoothness (Figure 2A). Irregularity on the corneal surface was significantly increased after the 7-day period of UVB exposure (group 2; Figure 2A-b and g), compared with the normal status of the blank control (group 1; Figure 2A-a and f). No evident corneal surface irregularity was observed in the groups protected with the Etafilcon A contact lenses (group 4; Figure 2A-d and i) or the HEMA+MA contact lenses (group 5; Figure 2A-e and j). However, significant corneal surface irregularity after UVB exposure was found in the group with Vifilcon A contact lenses. As for the corneal epithelial smoothness after UVB exposure, quantitative analysis showed a difference (Figure 2B), and the difference was in agreement with the extent of the corneal opacity. Severe corneal opacity was detected in the group without contact lens protection (group 2; Figure 2C-b and its corresponding negative image below), in contrast to the absence of corneal opacity in the blank control group (group 1; Figure 2C-a). Comparably, severe corneal opacity was also seen in the group with the Vifilcon A contact lenses (group 3; Figure 2C-c). Nevertheless, with protection by the Etafilcon A (group 4; Figure 2C-d) and the HEMA+MA contact lenses (group 5; Figure 2C-e), no UVB-induced corneal opacity was observed. The difference in corneal opacity can be better seen in the corresponding negative images (Figure 2C), which was also supported by quantitative analysis (Figure 2D).

With lissamine green staining, a large dark blue devitalized ocular surface area was easily identified in the cornea from the UVB group (group 2; Figure 2E-b and its corresponding negative image) and the group with the Vifilcon A contact lenses (group 3; Figure 2E-c). In contrast, no dark blue devitalized epithelial areas were found in the eyes from the blank control group (group 1; Figure 2E-a, and its negative image), or in the eyes protected with the Etafilcon A contact lenses (group 4; Figure 2E-d) and the HEMA+MA contact lenses (group 5; Figure 2E-e). The difference in the lissamine green staining was also indicated by quantitative analysis (Figure 2F).

### Ultraviolet-blocking contact lenses prevented thinning of the corneal epithelial layer after ultraviolet B irradiation

**Figure 3:**
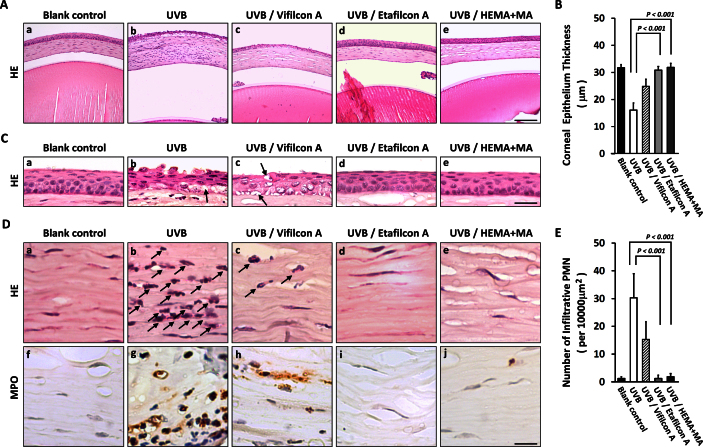
Histological assessments of in vivo protective efficacy by contact lenses. **A** and **C**: Hematoxylin and eosin (HE) staining of the corneas showed disordered stroma, a thinner epithelium layer, and more apoptotic vacuoles (indicated by arrows) in the ultraviolet B (UVB) and the Vifilcon A groups after UVB exposure, but not in the groups with UVB-blocking contact lenses (Etafilcon A and HEMA+MA) or in the blank control group. **B**: The reduction in the corneal epithelium thickness was significantly prevented in the groups with UVB-blocking contact lenses (Etafilcon A and HEMA+MA) compared to the UVB group (p<0.001 as determined wt the Wilcoxon-Mann–Whitney test; n=8). **D**: The corneal stroma showed infiltration of polymorphonuclear (PMN) leukocytes (indicated by arrows) after UVB irradiation, which was not seen in the eyes with UVB-blocking contact lenses (Etafilcon A and HEMA+MA). **E**: The infiltrative PMN leukocytes among the five study groups were shown by quantitative analysis (p<0.001 as determined by the Wilcoxon-Mann–Whitney test; n=5). The scale bar in **A**-e represents 100 μm, in **C**-e represents 30 μm, and in **D**-j represents 15 μm.

Histological analysis showed damage corresponding to the findings on the corneal surface. The thickness of the corneal epithelial layer was significantly reduced after UVB irradiation (group 2; Figure 3A-b) compared to that of the blank control group (group 1; Figure 3A-a). When the ocular surface was protected with Etafilcon A contact lenses (group 4; Figure 3 A-d) or HEMA+MA contact lenses (group 5; Figure 3A-e), the corneal epithelial thickness was maintained even after UVB irradiation. In contrast, no comparable protective effect was seen with the Vifilcon A contact lenses (group 3; Figure 3A-c). The mean central corneal epithelial thickness was 37.37±1.20 μm in the blank control group (group 1), 16.06±2.64 μm in the UVB group (group 2), 24.88±2.63 μm in the Vifilcon A group (group 3), 30.87±1.39 μm in the Etafilcon A group (group 4), and 31.85±1.54 μm in the HEMA+MA group (group 5). Quantitatively, the difference in corneal epithelial thickness was significant between the UVB group and the Etafilcon A group and the HEMA+MA group (p<0.001, Figure 3B). In addition, epithelial exfoliation was commonly found in the UVB group (group 2; Figure 3C-b), which was ameliorated in the Vifilcon A group (group 3; Figure 3C-c). The corneas covered with the Etafilcon A (group 4) and HEMA+MA (group 5) contact lenses did not show epithelial exfoliation and exhibited fewer vacuoles in the epithelium (Figure 3C-d and e). Whereas, many more vacuoles were found in the UVB group (arrow-indicated in Figure 3C-b) and the Vifilcon A group (arrow-indicated in Figure 3C-c).

### Less polymorphonuclear leukocyte infiltration under the protection of ultraviolet-blocking contact lenses

After UVB irradiation, the corneal stroma thickened due to edema, and infiltration of PMN leukocytes was observed (group 2; Figure 3A-b and arrow-indicated in D-b). In contrast, under the protection of the Etafilcon A contact lenses (group 4; Figure 3A-d and D-d) and the HEMA+MA contact lenses (group 5; Figure 3A-e and D-e), no evidence of edema was found in the stroma after UVB irradiation, and PMN leukocyte infiltration appeared to be completely prevented. In the group with the Vifilcon A contact lenses, although less edema was found, PMN leukocyte infiltration was only partly prevented (group 3; Figure 3A-c and arrow-indicated in D-c). Quantitatively, the density of the infiltrative PMN leukocytes in the groups with the Etafilcon A contact lenses (group 4) and the HEMA+MA contact lenses (group 5) was significantly decreased, compared to that of the UVB group (group 2; p<0.001, Figure 3E). To confirm this finding, the identity of the PMN leukocytes was confirmed with MPO expression as a marker. The results indicated infiltration of MPO^+^ cells into the stroma after UVB irradiation (group 2; Figure 3D-g), but not in the corneal stroma of the blank control (group 1; Figure 3D-f) or in the groups with UVB-blocking contact lenses (group 4 and group 5; Figure 3D-i and j).

### Corneal epithelial cell populations were maintained with ultraviolet B–blocking contact lenses after ultraviolet B irradiation

**Figure 4:**
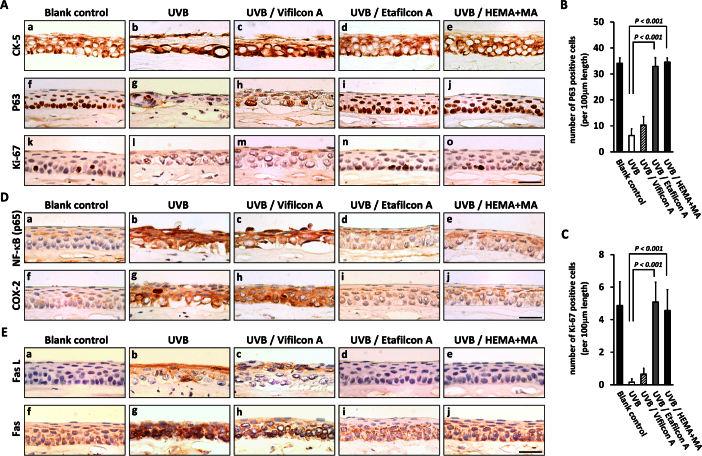
Ultraviolet B–induced proinflammatory and cell death signaling status among study groups. **A**: Immunohistochemical analysis showed severe loss of CK-5^+^ cells, P63^+^ cells, and Ki-67^+^ cells after ultraviolet B (UVB) irradiation, which was prevented by UVB-blocking contact lenses (Etafilcon A and HEMA+MA). **B** and **C**: Quantitative analysis of P63^+^ and Ki-67^+^ cells in the corneal epithelium among the five study groups was performed (p<0.001 as determined with the Wilcoxon-Mann–Whitney test; n=5). **D** and **E**: The expression of nuclear factor-kappa B (p65), cyclooxygenase-2, Fas L, and Fas was increased after UVB irradiation and was prevented by the UVB-blocking contact lenses (Etafilcon A and HEMA+MA). All scale bars represent 30 μm.

Since the corneal epithelial layer became thinner after UVB irradiation, it would be more informative to show which cell populations were damaged by UVB irradiation and whether all cell populations were protected by the UVB-blocking contact lenses. Following UVB irradiation, the CK-5^+^ epithelial cells were found disarranged (group 2; Figure 4A-b), and the P63^+^ basal cells became scarce (group 2; Figure 4A-g) compared to the status of the blank control (group 1; Figure 4A-a and f). Notably, the P63^+^ basal cells were found near the corneal surface, indicating substantial death of differentiating or terminally differentiated epithelial cells. In addition, the loss of proliferative basal cells was indicated by the lack of Ki-67^+^ cells after UVB irradiation (group 2; Figure 4A-l). The Vifilcon A contact lenses (group 3) had only limited cellular protective effects (Figure 4A-c, h and m), as the corneas covered by these lenses showed damage similar to that observed in the unprotected UVB group (group 2; Figure 4A-b, g and l). In contrast, the corneas in the groups covered with the UVB-blocking Etafilcon A contact lenses (group 4) or the HEMA+MA contact lenses (group 5) maintained normal cellular properties after UVB irradiation, as represented by better arrangement of the CK-5^+^ epithelial cells (Figure 4A-d and e), and maintenance of the P63^+^ basal cells and the Ki-67^+^ proliferative basal cells (Figure 4A-i, j, n, and o, respectively). Quantitative analysis showed significant maintenance of P63^+^ or Ki-67^+^ basal cell populations in the groups protected by the Etafilcon A contact lenses (group 4) and the HEMA+MA contact lenses (group 5), compared to the unprotected UVB group (group 2; p<0.001; Figure 4B,C).

### Inhibitory effects on proinflammatory factors by ultraviolet B–blocking contact lenses

To further understand the underlying mechanisms through which UVB-blocking contact lenses may ameliorate the symptoms of UVB-induced photokeratitis, immunohistochemical staining was performed to examine the status of four proinflammatory factors, including NF-κB (p65), COX-2, Fas, and Fas L (Figure 4D and E). High levels of NF-κB (p65; Figure 4D-b), COX-2 (Figure 4D-g), Fas L (Figure 4E-b), and Fas (Figure 4E-g) expression were seen in the corneas exposed to UVB (group 2), in contrast to the minimal or absent expression in the corneas of the blank group (Figure 4D-a, f and Figure 4E-a, f). Notably, with the protection of the UVB-blocking Etafilcon A contact lenses (group 4; Figure 4D-d, i and Figure 4E-d, i) and the HEMA+MA contact lenses (group 5; Figure 4D-e, j and Figure 4E-e, j), all four proinflammatory factors were attenuated. In contrast, the corneas with the Vifilcon A contact lenses (group 3) did not show evident attenuation of the four proinflammatory factors (Figure 4D-c, h and Figure 4E-c, h), since they were expressed to a level close to that found in the UVB group.

## Discussion

### Ultraviolet-induced photodamage and novel insight of corneal protection provided by ultraviolet-blocking contact lenses

UV irradiation is the major environmental risk factor to human eyes [1-4], causing damage that leads to the pathogenesis of photokeratitis, pterygium, iritis, and cataract. In recent years, a great deal of evidence has supported the belief that UVB-activated inflammatory factors play important roles in the initiation and progression of ocular surface and anterior chamber diseases [9,20,22,23]. In particular, corneal surface damage and inflammatory cellular responses induced by UVB irradiation are of major concern, due to the unavoidable and highly susceptible nature of the cornea to UVB exposure. For protection against UV-induced photodamage, UV-blocking contact lenses may be more advantageous than framed lenses, particularly for preventing peripheral light to focus on the central cornea [14,15]. However, few previous reports have addressed the UV-protective effects of contact lenses under in vivo conditions.

Although the beneficial effects of some UV-blocking contact lenses, the Senofilcon A contact lens, for example, have been investigated in several population-based studies [1,24,25] as well as in laboratory animal models [17-19], the present study provides significant novel insights not addressed in previous reports. First, the underlying mechanism in which UV-blocking contact lenses may protect against UVB-induced photodamage has not been described in terms of changes in corneal cell populations. In our mouse model, the thinning of the corneal epithelial layer after UVB irradiation was caused by substantial cell death in all corneal epithelial populations, including P63^+^ basal cells, Ki-67^+^ proliferative basal cells, and CK-5^+^ differentiated cells. Our data also showed that the P63^+^ basal cells were the last epithelial cell population to survive after the photodamage caused by UVB irradiation for a substantial period, as exemplified by the 7-day period in our mouse model. In contrast, when the corneas were covered with UVB-blocking Etafilcon A contact lenses or HEMA+MA contact lenses, all epithelial cell populations maintained normal cellular properties after UVB irradiation. Corneal epithelial cells with more differentiated characteristics or those undergoing differentiation are likely more vulnerable to photodamage caused by UVB irradiation. Our results also demonstrated that UVB-blocking Etafilcon A contact lenses and HEMA+MA contact lenses provide protective effects on all three epithelial cell populations identified by their respective markers. Second, if UV-blocking contact lenses are effective for protection against UVB-induced photodamage, such protective effects have to persist long enough to be of positive clinical significance. This is particularly important for weekly contact lenses and even more important for monthly contact lenses. In the present study, we demonstrated that Etafilcon A and HEMA+MA weekly disposable contact lenses prevent UVB-induced photodamage under in vivo conditions even after the 7-day UVB irradiation protocol. This UV proof aspect has to be incorporated as a standard procedure for contact lens design, particularly for contact lenses intended for long-term wear. Third, it had been reported that the maximal amount of UVB reaching the human cornea from sunlight could reach approximately 0.18 J/cm^2^ per hour [26]. The daily UVB dose used in the present study was 0.72 J/cm^2^, equivalent to 4 h of daily sunlight exposure. In many circumstances, this UVB dose is close to daily exposure in the real lives of many people. To some professionals, such as lifeguards, this high dose of UVB exposure is a daily routine. Therefore, results of the present study indicate that the use of proper UV-blocking devices can never be overemphasized. Fourth, the percentage of UVB transmittance for each type of contact lens used in this study increased over the 7-day study period with the present experimental protocol. Since the UVB dosage used in this study was equivalent to 4 h of daily sunlight exposure [26], the potential loss of UVB absorption capacity after long-term contact lens wear has to be considered, particularly for monthly or longer users. Fifth, our data demonstrated that the extent of UVB transmittance was determinative in the resultant photodamage on the corneal epithelium, which may be used as a primary quality control measurement for contact lens manufacturers.

### Potential link between ultraviolet exposure and short-term visual performance

Clinically, the corneal surface topographic deviation in different orientations constitutes the main cause of irregular astigmatism [27,28]. Based on topographic changes, we developed a technique for analyzing the deviations on the corneal surface following the 7-day period of UVB irradiation. Our results demonstrated roughness or irregularity on the corneal surface (Figure 2A-g and h), suggesting that UVB irradiation might affect clinical visual performance. Thus, UV protection is important not only for corneal health but also for visual performance. Currently, clinical practice does not seem to emphasize a potential link between the extent of UV or sunlight exposure and the short-term adverse effect on visual performance. Our results in this study suggest that this important issue should be further explored in research and clinical practice.

### Ultraviolet-blocking contact lenses prevented the activation of inflammatory factors

The molecular mechanisms underlying the UV-blocking effects by contact lenses for the attenuation of apoptotic and inflammatory activities have not previously been fully elucidated. Because NF-κB activation plays a central role in the inflammatory response, growth-modulatory activities, cellular transformation, and cell death processes [29,30], UV-blocking contact lenses likely protect against photodamage primarily through inhibition of the NF-κB pathway. Many lines of evidence have been reported in favor of this possibility. For example, researchers have been shown that NF-κB mediates UV-induced release of interleukin-1, interleukin-6, and tumor necrosis factor-α from cultured human corneal epithelial cells [23]. It is therefore possible that UVB-induced NF-κB activation leads to the release of cytokines that initiate the photodamage processes, leading to the degeneration of the corneal epithelium. In addition, the activated NF-κB (p65), if being translocated into the nucleus, will facilitate transcription of many downstream genes, including COX-2, Fas, and Fas L [31], which are key mediators in recruiting inflammatory cells and cellular death progression [32-35]. In the present study, by using UVB-blocking contact lenses (Etafilcon A and HEMA+MA), but not non-UVB-blocking contact lenses (Vifilcon A), we demonstrated that the UVB-induced aberrant expression of inflammatory factors, including NF-κB (p65), could be inhibited (Figure 4D). Previous reports also showed that inhibition of NF-κB (p65) and COX-2 protects against oxidative and inflammatory corneal epithelial damage caused by UVB irradiation under in vitro and in vivo conditions [9,36,37]. Additionally, activation of the Fas-Fas L system, downstream of NF-κB (p65), has also been reported to play an important role in apoptosis of corneal cells after UVB exposure in the rabbit [12]. Therefore, the NF-κB pathway likely plays a pivotal role in UVB-induced photokeratitis, and the biologic efficacy of UVB-blocking contact lenses is achieved mainly though inhibition of the NF-κB pathway.

### Limitations of the present ultraviolet B mouse model

Although the results of the present study have exemplified the use of mouse model for assessing the efficacy of protection by contact lens against UVB, the present model has limitations. First, the mouse is a nocturnal animal and therefore contains a relatively low concentration of ascorbate in the corneal epithelium for UVB absorption compared with a diurnal animal. For example, the level of ascorbate in the rat corneal epithelium is 8 times lower than that in humans [38,39]. Although the ascorbate content represents only a fraction of the total antioxidant or UV absorption capacity within the corneal epithelium, it is highly likely that diurnal animals are equipped with a much better total protective mechanism against UV-induced damage than the nocturnal animals. Thus, the rodent corneal epithelium would be expected to be damaged by a substantially lower dose of UVB radiation, compared to that for a diurnal species. The UVB dose used in the present study may not cause comparable damage in the human corneal epithelium. However, to a small subgroup of the human population such as professionals routinely exposed to high UV irradiation at high altitudes or for those who have to work in sunshine on a long-term basis, even with the current UVB dose used in the present study, the potential damage remains to be cautiously considered. Second, the photokeratitis observed in the mouse UVB exposure model was induced by daily 90 s UVB exposure to reach a total amount of 0.72 J/cm^2^/day for a 7-day period. The experimental protocol allowed no time for the deletion of oxidants and inflammatory factors induced by such high phototoxic energy or enough time for the repair of the corneal epithelium. Therefore, the damage following UVB exposure represents only acute photokeratitis conditions. For assessment against chronic photokeratitis conditions, the experimental protocol has to be modified.

### Conclusions

Disposable soft contact lenses are used by millions of people around the world for vision improvement, protection, or cosmetic purposes. Thus, the efficacy of the UV-blocking properties of contact lenses will always be a major concern. Particularly, the biologic efficacy under in vivo conditions will be more informative than physical or in vitro assessments. The mouse model used in our study may act as a platform for assessing in vivo biologic efficacy for new UV-blocking materials intended for manufacturing contact lenses.
